# Anthocyanin-Rich Blackcurrant Pomace Mitigates Oxidative Stress and Affects Steroid Metabolism in the Testes of Rats Exposed to Silver Nanoparticles

**DOI:** 10.3390/nu17243809

**Published:** 2025-12-05

**Authors:** Michał Oczkowski, Katarzyna Dziendzikowska, Marcin Kruszewski, Joanna Gromadzka-Ostrowska, Agnieszka Grzelak

**Affiliations:** 1 Department of Dietetics, Institute of Human Nutrition Sciences, Warsaw University of Life Sciences (SGGW-WULS), 159c Nowoursynowska St., 02-776 Warsaw, Poland; katarzyna_dziendzikowska@sggw.edu.pl (K.D.); joanna_gromadzka-ostrowska@sggw.edu.pl (J.G.-O.); 2 Centre for Radiobiology and Biological Dosimetry, Institute of Nuclear Chemistry and Technology, 16 Dorodna Str., 03-195 Warsaw, Poland; m.kruszewski@ichtj.waw.pl; 3 Department of Molecular Biology and Translational Research, Institute of Rural Health, 2 Jaczewskiego Str., 20-090 Lublin, Poland; 4 Centre for Digital Biology and Biomedical Science-Biobank Lodz, Faculty of Biology and Environmental Protection, University of Lodz, 139 Pomorska Str., 91-402 Lodz, Poland; agnieszka.grzelak@biol.uni.lodz.pl

**Keywords:** blackcurrant, silver nanoparticles, oxidative stress, rats, testes, steroid metabolism

## Abstract

**Background/Objectives**: Silver nanoparticles (AgNPs), used in industry and medicine, can have a negative impact on the human organism, particularly on the reproductive system, while polyphenolic supplementation may reduce oxidative stress (OS) and enhance male reproductive potential. The aim of this study was to investigate the effects of anthocyanin-rich blackcurrant pomace (BC) on sex steroid hormone metabolism and the OS indicator in the testes of rats following exposure to AgNPs. **Methods**: Adult rats were fed with a control feed (CTR) or diet supplemented with a 2% BC (BC group). The rats from AgNano and AgNano+BC groups were treated with 20 nm AgNPs (30 mg/kg/day for 28 days by gavage). **Results**: The plasma testosterone (T) and plasma dihydrotestosterone (DHT) concentrations were decreased in all experimental groups compared to the control (CTR) animals. The co-treatment of animals with AgNPs and BC resulted in decreased oestrogen receptor (ESR2) levels in the testes as compared to rats fed with a diet with BC alone, and the up-regulation of mRNA level of genes involved in T synthesis and metabolism (*StAr*, *Cyp11a1*, *Hsd17b3*, *Hsd3b3*, *Cyp19a1*, and *Srd5a1*), and steroid hormone signalling (AR, ESR1, and ESR2) compared to the Ctr group. The addition of BC to the diet of rats treated with AgNPs resulted in decreased protein carbonyls in the testes as compared to AgNPs-treated animals. **Conclusions**: The study demonstrated that relatively low AgNPs administration to rats was associated with increased oxidative stress in the gonads. Incorporating BC into the animals’ feed mitigated AgNPs-induced oxidative stress and stimulated the expression of genes involved in steroid synthesis and metabolism in testes. The bioactive compounds in blackcurrant pomace have plausible mechanisms to influence reproductive health.

## 1. Introduction

Male infertility is a growing global health problem in many countries. The percentage of men who have not been able to conceive accounts for 30% to 70% of infertility cases, depending on the geographic region. Overall, 56 million cases were reported globally in 2019 [1,2]. The male infertility prevalence coincides with lifestyle factors [3,4] and chronic inflammatory diseases [5]. Furthermore, exposure to various environmental contaminants significantly contributes to a decline in male reproductive potential [6]. Recent evidence suggests that engineered nanoparticles (ENPs) may also impair male fertility [7,8].

Silver nanoparticles (AgNPs) are the most widely used ENPs due to their unique antimicrobial properties, enabling diverse applications across medicine, pharmacy, cosmetics, household chemicals, electronics, agriculture, food production, and textiles [9]. In the food industry, AgNPs are used primarily as an antimicrobial agent in food packaging, preservatives, fragrances, and colourants [10]. Despite the growing use of AgNPs in consumer products, their potential toxicity remains insufficiently evaluated. In vitro and animal model studies suggest that AgNPs may adversely affect human health, with effects depending on exposure duration, and NPs’ size, shape, and surface modifications [11]. Regardless of the route of administration, exposure to AgNPs leads to oxidative cellular damage [12] and inflammatory response [13]. The findings from in vivo and in vitro models have also shown that AgNPs negatively affect the reproductive system, with toxicity depending on dose, duration of exposure, and nanoparticle properties, such as size and surface modifications [14,15], with oxidative stress being a leading mechanism in AgNPs-induced reproductive toxicity [16].

Given this, dietary anti-oxidants may exhibit potential protective effects. Research shows that a diet rich in anti-oxidant plant compounds, such as polyphenols, can reduce oxidative stress in the body and provide anti-inflammatory benefits [17]. The plant-derived compounds with high anti-oxidant potential appear to be a straightforward and practical approach that may increase male reproductive potential [18,19].

Fruit pomace, a byproduct of juice production, is a rich source of polyphenolic compounds that can be utilised as a functional food ingredient with significant health benefits [20]. Blackcurrant pomace (BC), in particular, contains high levels of anthocyanins, ranging from 344 to 1046 mg per 100 g of pomace [21], and exhibits significant anti-oxidant potential and estrogenic properties [22]. In vivo studies [23] indicate that fruit extracts can improve sperm quality, maintain blood–testis barrier integrity, and regulate hormonal balance. Moreover, anthocyanins have been shown to activate antioxidative pathways in the reproductive system [24].

Therefore, this study aimed to investigate the sub-chronic effects of anthocyanin-rich blackcurrant pomace (BC) as an additive to the diet of animals on steroid hormone metabolism and oxidative stress indicators in the testes of rats exposed to 20 nm bovine serum albumin-coated AgNPs. We applied an AgNPs dose (30 mg/kg/day for oral exposure) that corresponds to the oral no-observed-adverse-effect level (NOAEL) established in previous sub-chronic in vivo toxicity studies in rats [25,26]. When adjusted from rats to humans based on body area, this represents an equivalent exposure of 5 mg/kg/day [27], Such a level may reflect the ongoing and anticipated rise in the use of AgNP-containing products. This trend is expected to intensify, particularly with the introduction of new food items and food contact materials incorporating AgNPs [28].

## 2. Materials and Methods

### 2.1. Materials

All the chemicals were obtained from Sigma-Aldrich (St. Louis, MO, USA) or from Life Technologies (Waltham, MA, USA) (the chemicals for molecular biology). The spherical 20 nm silver nanoparticles (AgNPs) were acquired form PlasmaChem^®^ (Berlin, Germany). Isoflurane was purchased from Baxter Healthcare, Warsaw, Poland. The diagnostic kits for alanine (ALT) and aspartate (AST) aminotransferases were obtained from Human Gesellschaft für Biochemica und Diagnostica GmbH (Wiesbaden, Germany). The ELISA assays were obtained from Wuxi Donglin Sci & Tech Development Co., Ltd. (Wuxi, China) for LH, from Demeditec Diagnostics GmbH (Kiel, Germany) for steroid hormones, and from EIAab^®^ (Wuhan, China), for ESR1, ESR2, AR, and Aro determinations.

### 2.2. Characterisation of Silver Nanoparticles

The spherical AgNPs, with a nominal diameter of 20 ± 5 nm, were obtained from PlasmaChem (Berlin, Germany) and prepared according to the protocol previously published [29]. The detailed characterisation of AgNPs and the preparation of a working solution have been described previously [29,30,31] and are summarised in Table 1. The images of AgNPs have already been published by Lankoff et al. [29] and Dziendzikowska et al. [30]. To obtain a working solution, 5 mg of AgNPs were dispersed in 800 µL of distilled water and sonicated for three minutes, using a total ultrasound energy of 420 J/m^3^. Immediately after sonication, 100 µL of 10× concentrated phosphate-buffered saline (PBS, Sigma-Aldrich, St. Louis, MO, USA) and 100 µL of 15% bovine serum albumin (BSA, Sigma-Aldrich, St. Louis, MO, USA) were added to the suspension. A fresh suspension of AgNPs was prepared each time shortly before administration to animals.

### 2.3. Animals

The in vivo experiment was conducted under procedures approved by the 3rd Local Ethical Commission at Warsaw University of Life Sciences (WULS-SGGW), Warsaw, Poland (Resolution No 71/2013 from 19 December 2013), in compliance with UE Directive (2010/63/UE), Polish law, and the 3R rules (Replacement, Reduction, and Refinement) and according to ARRIVE guidelines.

Adult male Fischer344 rats (F344/DuCrl) (*n* = 28; initial body weight: 296.8 ± 3.7 g) were obtained from Charles River Laboratories, Inc. (Sulzfeld, Germany). The rats underwent a one-week acclimatisation period. Following this, they were randomly assigned to four experimental groups (one group per cage) based on the treatment with silver nanoparticles (AgNPs) and dietary intervention, using a randomised block design according to the rats’ body weights (see Table 2).

The animals from the BC group were fed pellets (AIN-93, Zoolab, Sędziszów, Poland), providing the rats’ nutritional requirements [32], supplemented with 2% (*w*/*w*) BC. The basic characteristics of the phenolic content in the animal feed were presented in a published paper [33]. The analysis of selected flavonoids and phenolic acids in BC demonstrated that more than 90% (*w*/*w*) of the analysed compounds were anthocyanins. The characteristic of phenolics in experimental feed is presented in Table S1 (in Supplementary Materials). Rats in the AgNano group were administered 20 nm AgNPs (30 mg/kg bw, dissolved in 200 μL of distilled water) daily by gastric gavage for 28 days. Rats in the AgNano + BC group received both treatments simultaneously. The number of animals in the experimental groups was determined based on the literature data from [34]. Additionally, based on the results from the G*Power (version 3.1.9.7) software (a priori power analysis test) and data from a previous in vivo study (plasma testosterone level as the primary outcome), it was confirmed that seven animals per group, across four experimental groups, adequately support the experiment.

The rats’ health, behaviour (signs of pain or distress, or unusual appearance), and food and water consumption were monitored daily. No humane endpoints occurred during the experiment. To minimise the stress of the animals, the number of personnel involved in the experiment was reduced to a minimum throughout the experiment. The handling of animals was always carried out by the same trained staff at the same time each day.

After 28 days of experimentation, the rats were anaesthetised with isoflurane (Baxter Healthcare, Warsaw, Poland) and exsanguinated via the left ventricle of the heart. Blood was collected into tubes coated with EDTA (ethylenediaminetetraacetic acid) and then centrifuged at 2300× *g* (3500 rpm) at 4 °C for 20 min to isolate plasma samples. The testes were carefully dissected, rinsed with ice-cold PBS, dried on filter paper, weighed, and immediately frozen in liquid nitrogen. Plasma and testis samples were stored at −80 °C for further biochemical analyses.

### 2.4. Plasma and Liver Biochemical Indices of the Physiological Status of Animals

To evaluate the physiological status of animals, the activities of alanine aminotransferase (ALT) and aspartate aminotransferase (AST) in plasma and liver homogenates were evaluated. The ALT and AST activities were analysed according to the kinetic method recommended by the Expert Panel of the IFCC (International Federation of Clinical Chemistry, Milan, Italy) using commercially available kits (Human Gesellschaft für Biochemica und Diagnostica GmbH, Wiesbaden, Germany), according to the manufacturer’s protocol.

The liver homogenates were prepared by the homogenization of tissue samples in 0.01 M phosphate buffer (PBS; pH 7.4) containing 0.0027 M potassium chloride, 0.137 M sodium chloride, and 1 mM of EDTA (all reagents were purchased from Sigma-Aldrich, St. Louis, MO, USA) at a volume of ratio tissue: buffer of 1:7, using a motorised homogenizer (Bio-Gen PRO 200, PRO Scientific, Oxford, CT, USA). The homogenates were centrifuged at 10,000× *g* for 15 min at 4 °C using a Multifuge 3L-R (Kendro, Asheville, NC, USA), and the supernatant was used in further analyses. The liver’s ALT and AST activity was determined using commercially available kits (Human Gesellschaft für Biochemica und Diagnostica GmbH, Wiesbaden, Germany), according to the manufacturer’s protocol.

### 2.5. Assessment of the Reproductive Hormone Concentrations

The plasma LH concentration was determined using ELISA assay (cat. no. DL-LH-Ra, Wuxi Donglin Sci & Tech Development Co., Ltd., Wuxi, China). The assay detection range was 370.4 to 30,000 pg/mL. The minimum detectable level of LH was below 135.7 pg/mL, and the intra-assay and inter-assay precision were below 10% and 12%, respectively. All samples were analysed in duplicate.

The steroid hormone levels in testes were analysed following the extraction method previously described by Dziendzikowska et al. [35]. The concentrations of testosterone (T), dihydrotestosterone (DHT), and 17β-estradiol (E2) were then determined using ELISA kits (Demeditec Diagnostics GmbH, Kiel, Germany; cat. No: DE1559, DE2330, and DE43399, respectively), following the manufacturer’s guidelines. The sensitivity thresholds for the assays were 0.083 ng/mL for T, 7.23 pg/mL for DHT, and less than 1.399 pg/mL for E2.

### 2.6. Protein Levels of Oestrogen (ESR1 and ESR2), Androgen Receptors (AR), and Aromatase (Aro) in the Testis

Tissue samples intended for the analysis were homogenised in cold phosphate-buffered saline (PBS, pH 7.4, Sigma-Aldrich, St. Louis, MO, USA). After two cycles of freezing and thawing at 4 °C, the homogenates were centrifuged at 5000× *g* (4 °C), and the supernatants were collected for further analysis. All samples were analysed in duplicate. Levels of ESR1, ESR2, AR, and Aro were quantified using ELISA assay (EIAab^®^, Wuhan, China; kits Cat. No: E1050r, E2300r, E1252r, and E2098r, respectively). The detection ranges for ESR1, ESR2, Aro, and AR were 0.31–20.0 ng/mL, 0.15–10.0 ng/mL, 78.0–5000 pg/mL, and 0.12–8.00 ng/mL, respectively.

### 2.7. Gonadal Steroidogenesis Gene Expression Analysis

Total RNA was isolated from the testis samples using the MagNA Pure LC 2.0 Automated Sample Preparation DNA/RNA Instrument (Roche, Basel, Switzerland) according to the manufacturer’s protocol. Genomic DNA was removed through DNase I digestion (RNase-free DNase, Life Technologies, Grand Island, NY, USA). Subsequently, 1 µg of total RNA was reverse transcribed with the SuperScript™ III First-Strand Synthesis SuperMix (Life Technologies, ThermoFisher, Waltham, MA, USA). Quantitative PCR (qPCR) analysis was conducted using the C1000 Thermal Cycler–CFX96 Real-Time System (Bio-Rad, Hercules, CA, USA). Primer sequences for RT-qPCR (Table 3) were purchased from Genomed (Warsaw, Poland). The subsequent steps of analysis, including the RT-qPCR mixture and setup, were performed according to the method described in a previously published paper [36]. The ACTB and GAPDH were used as reference genes. The RT-qPCR data were normalised by calculating the geometric mean of the reference genes, using the negative control to standardise relative expression levels, and then assessing whether significant differences existed between the control and experimental groups. After entering multiple target and reference genes, the software used the normalised target gene values to show how the groups differed, and provided the corresponding *p*-value [37]. To normalise the relative gene expression, the gene expression in the control group was set to one. The significance was tested via Pair-wise Fixed Reallocation Randomisation Test using REST 2009 version 1 software [38,39].

### 2.8. Oxidative Stress Parameters in Testes

The protein carbonyls levels in testis homogenates were evaluated as marker of oxidative stress using a colorimetric assay kit (Cayman Chemical Company, Ann Arbor, MI, USA; Cat. No: 10005020), according to the manufacturer’s instructions. The analyses were performed in duplicates.

### 2.9. Statistical Analysis

Statistical analyses were performed using Statistica software version 13.3 (TIBCO Software Inc., San Ramon, CA, USA). All results were presented as mean ± S.E.M. (standard error of the mean). A one-way analysis of variance (ANOVA) with Duncan’s post hoc test was used to examine differences between animal groups. The differences were considered statistically significant at *p* < 0.05. Gene expression levels were calculated as the fold change in relation to the control (CTR) group. Significance was tested using the Pair-Wise Fixed Reallocation Randomisation Test in REST 2009 version 1 software.

## 3. Results

### 3.1. Rats’ Weight Gain, Testis Weight, Gonadosomatic Index, and Food Intake

The final body weight, total weight gain, testis weight, and average daily food intake did not change significantly among the experimental groups (ANOVA: NS; Table 4). While the final body weights were comparable across all animal groups, the gonadosomatic index (GSI) was significantly higher in the BC group compared to the AgNano and control (CTR) groups (*p* = 0.022 and *p* = 0.018, respectively).

### 3.2. Plasma and Liver Alanine and Aspartate Aminotransferases Activities

The analysis of ALT and AST activity is presented in Table 5. The analysis revealed that none of the tested parameters differed significantly, neither in the plasma nor in liver homogenate (ANOVA, NS).

### 3.3. Plasma Hormone Concentrations

**Figure 1:**
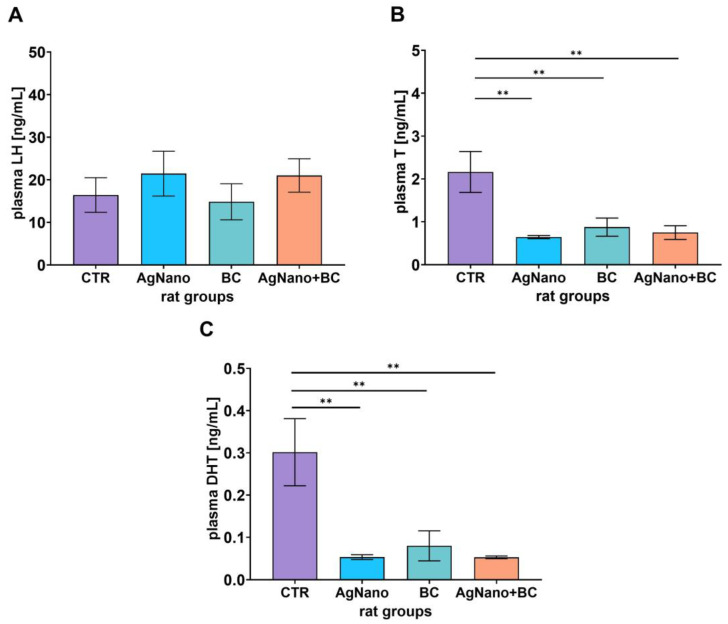
Plasma concentration of luteinizing hormone (LH, (**A**)), testosterone (T, (**B**)), and dihydrotestosterone (DHT, (**C**)) in rats. ANOVA, plasma T conc.: *p* = 0.0023, η_p_^2^ = 0.460; (**B**); ANOVA, plasma DHT conc.: *p* = 0.0014, effect size η_p_^2^ = 0.483; ** *p* < 0.01, one-way ANOVA with Duncan’s post hoc test; animals. Data were expressed as mean ± S.E.M. CTR—control group; AgNano—rats exposed to AgNPs (30 mg/kg/day for 28 days, by gavage; BC—rats receiving diet containing blackcurrant pomace (2% m/m); and AgNano + BC, rats exposed to AgNPs and fed with diet containing blackcurrant pomace.

The concentrations of luteinizing hormone (LH), testosterone (T), and dihydrotestosterone (DHT) in plasma are illustrated in Figure 1A–C. No statistically significant differences were found in the concentration of plasma LH among the experimental groups (ANOVA, *p* = 0.651; effect size, η_p_^2^ = 0.067; Figure 1A). The mean LH plasma concentrations in CTR, BC, AgNano, and AgNano + BC groups were 16.43 ng/mL (95% CI [6.48–26.37]), 14.84 ng/mL (95% CI [4.47–25.20]), 21.45 ng/mL (95% CI [8.56–34.34]), and 21.01 ng/mL (95% CI [10.91–31.11]), respectively. In contrast, the plasma levels of T and DHT were significantly reduced in the BC (0.877 ng/mL (95% CI [0.360–1.394]) and 0.080 ng/mL (95% CI [−0.007–0.167])), AgNano (0.644 ng/mL (95% CI [0.556–0.732]) and 0.053 ng/mL (95% CI [0.039–0.067])), and AgNano + BC (0.750 ng/mL (95% CI [0.339–1.161]) and 0.052 ng/mL (95% CI [0.045–0.061])) groups when compared to the control group (2.163 ng/mL (95% CI 0.995–3.330]) and 0.302 ng/mL (95% CI [0.045–0.061]), respectively); *p* = 0.0039 and *p* = 0.0023, *p* = 0.0016 and *p* = 0.0012, *p* = 0.0024 and *p* = 0.0014, respectively; Figure 1B,C. In the CRT group, the plasma T concentration was highest at 2.16 ng/mL (95% CI [1.00–3.33]). In turn, the mean plasma T concentration in the BC group was at 0.88 ng/mL (95% CI [0.36–1.39]), whereas the AgNano and NPs + BC groups presented mean plasma T levels at 0.64 ng/mL (95% CI [0.56–0.73]) and 0.75 ng/mL (95% CI [0.34–1.16]), respectively (Figure 1B). Similar changes were observed in relation to the plasma DHT concentration. The highest concentration was observed at 0.301 ng/mL (95% CI [0.107–0.496]). In groups BC, AgNano, and AgNano + BC, the mean plasma DHT concentrations were 0.080 ng/mL (95% CI [−0.007–0.167]), 0.053 ng/mL (95% CI [0.039–0.067]), and 0.053 ng/mL (95% CI [0.045–0.061]).

### 3.4. Steroid Hormones and Aromatase Levels in the Testes

**Figure 2:**
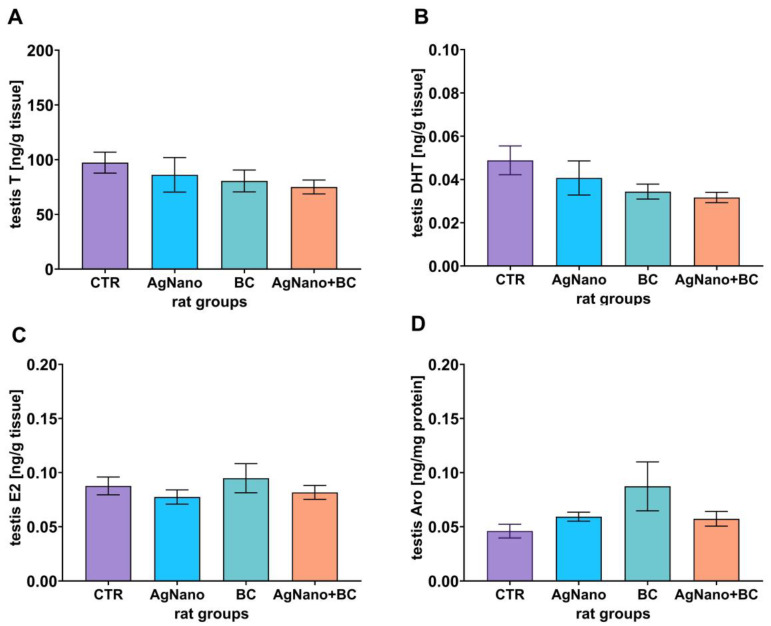
The levels of testosterone (T, (**A**)), dihydrotestosterone (DHT, (**B**)), 17β-estradiol (E2, (**C**)), and aromatase (Aro, (**D**)) in rat testes. One-way ANOVA with Duncan’s post hoc test. Data were expressed as mean ± S.E.M. CTR—control group; AgNano—rats exposed to AgNPs (30 mg/kg/day for 28 days, by gavage; BC—rats receiving diet containing blackcurrant pomace (2% m/m); and AgNano + BC, rats exposed to AgNPs and fed with diet containing blackcurrant pomace.

As illustrated in Figure 2A–C, the testicular levels of T, DHT, and 17β-estradiol (E2) remained consistent across all experimental groups (ANOVA, *p* = 0.592; η_p_^2^ = 0.090, 0.218, η_p_^2^ = 0.194, and *p* = 0.526, η_p_^2^ = 0.103 for T, DHT, and Aro, respectively; Figure 2A–C). No significant differences were also found in the Aro protein level among the groups (ANOVA, *p* = 0.121, η_p_^2^ = 0.237; Figure 2D). The mean testicular T and DHT level across the experimental groups were 97.3 ng/mL (95% CI [72.66–121.93] and 0.049 ng/mL (95% CI [0.032–0.066], 86.2 ng/mL (95% CI [47.6–124.8]) and 0.041 ng/mL (95% CI [0.022–0.060]), 80.6 ng/mL (95% CI [52.8–108.4]) and 0.034 ng/mL (95% CI [0.025–0.044]), and 75.1 ng/mL (95% CI [58.7–91.4]) and 0.032 ng/mL (95% CI [0.025–0.038]) in the CTR, AgNano, BC, and AgNano + BC groups, respectively. At the same time, the mean testicular E2 and Aro were at 0.088 ng/mL (95% CI [0.067–0.109]) and 0.046 ng/mg protein (95% CI [0.031–0.061]), 0.077 ng/mL (95% CI [0.061–0.093]) and 0.059 ng/mg protein (95% CI [0.049–0.069]), 0.095 ng/mL (95% CI [0.058–0.132]) and 0.087 ng/mg protein (95% CI [0.029–0.146]), and 0.081 ng/mL (95% CI [0.065–0.098]), and 0.057 ng/mg protein (95% CI [0.038–0.076]) in the CTR, AgNano, BC, and AgNano + BC groups, respectively.

### 3.5. Oestrogen and Androgen Receptor Protein Levels in the Testes

**Figure 3:**
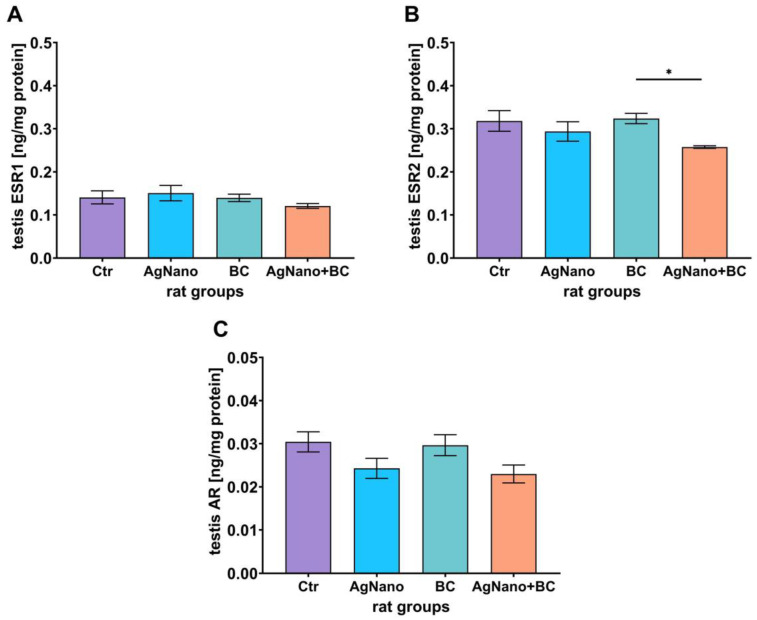
Steroid receptor protein level of oestrogen receptor type 1 (ESR1, (**A**)), oestrogen receptor type 2 (ESR2, (**B**)), and androgen receptor (AR, (**C**)). One-way ANOVA with Duncan’s post hoc test. * *p* < 0.05. Data were expressed as mean ± S.E.M. CTR—control group; AgNano—rats exposed to AgNPs (30 mg/kg/day for 28 days, by gavage); BC—rats receiving diet containing blackcurrant pomace (2% m/m); and AgNano + BC, rats exposed to AgNPs and fed with diet containing blackcurrant pomace.

The level of oestrogen receptor type 1 (ESR1) and androgen receptor (AR) in the testes did not differ significantly between the experimental groups (ANOVA, *p* = 0.528, η_p_^2^ = 0.103, Figure 3A, and *p* = 0.090, η_p_^2^ = 0.261, Figure 3C, for ESR1 and AR, respectively). The mean levels of analysed proteins in the rats’ testes were 0.141 ng/mg protein (95% CI [0.102–0.180]) and 0.30 ng/mg protein (95% CI [0.024–0.036]), 0.140 ng/mg protein (95% CI [0.107–0.194]) and 0.030 ng/mg protein (95% CI [0.019–0.030]), 0.151 ng/mg protein (95% CI [0.117–0.162]) and 0.024 ng/mg protein (95% CI [0.023–0.036]), and 0.120 ng/mg protein (95% CI [0.106–0.136]) and 0.023 ng/mg protein (95% CI [0.017–0.028]) in the CTR, AgNano, BC, and AgNano + BC groups, respectively. The post hoc analysis revealed a significant reduction in oestrogen receptor type 2 (ESR2) protein level in the AgNano + BC group compared to the BC group (*p* = 0.039; 0.258 ng/mg protein (95% CI [0.249–0.266]) versus 0.324 ng/mg protein (95% CI [0.293–0.354]), Figure 3B). The mean levels of ESR2 in the CTR and AgNano groups were 0.318 ng/mg protein (95% CI [0.260–0.377] and 0.294 ng/mg protein (95% CI [0.238–0.350], respectively.

### 3.6. Analysis of Gene Expression in the Testis

**Figure 4:**
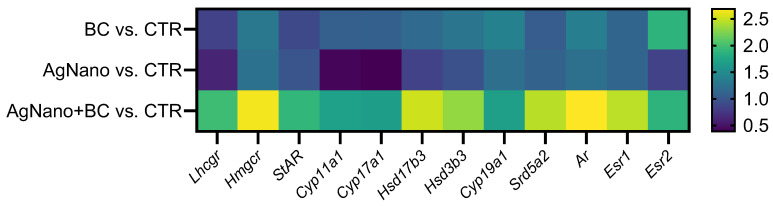
Heatmap representing the analysed gene expressions.

The gene expression analysis conducted in the testis revealed distinct patterns of up-regulation and down-regulation across experimental groups, highlighting the effect of treatment on various biological pathways. As shown in Figure 4 and in Table S2 (in Supplementary Material), in the testes, the co-treatment of animals with AgNano and BC resulted in the up-regulation of most of the analysed genes compared to the control group. Essentially, the up-regulated genes included those involved in regulating the hypothalamic–pituitary–gonadal axis (*Lhcgr*), cholesterol synthesis (*Hmcgr*), and testosterone synthesis and metabolism (*StAR*, *Cyp11a1*, *Hsd17b3*, *Hsd3b3*, *Cyp19a1*, and *Srd5a1*). Additionally, genes involved in steroid hormone signalling (*Ar*, *Esr1*, and *Esr2*) were also up-regulated. In contrast, two genes (*Cyp11a1* and *Cyp17a1*) were down-regulated in the AgNano group compared to the control animals.

### 3.7. Oxidative Stress in the Testes

**Figure 5:**
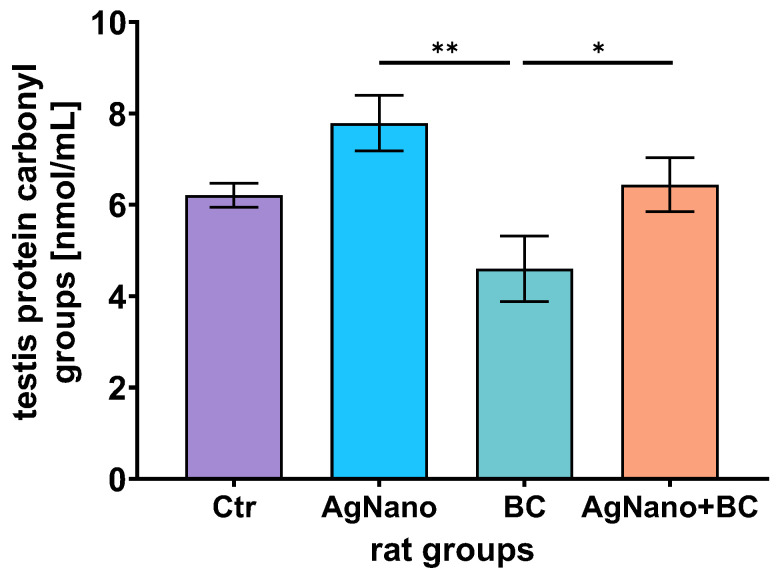
Protein carbonyl group concentration in rat testes. One-way ANOVA with Duncan’s post hoc test. ANOVA, *p* = 0.0081, η_p_^2^ = 0.408. * *p* < 0.05, ** *p* < 0.01. Data were expressed as mean ± S.E.M. CTR—control group; AgNano—rats exposed to AgNPs (30 mg/kg/day for 28 days, by gavage); BC—rats receiving diet containing blackcurrant pomace (2% m/m); and AgNano + BC—rats exposed to AgNPs and fed with diet containing blackcurrant pomace.

The results presented in Figure 5 demonstrated statistically higher concentrations of protein carbonyl in the AgNano (*p* = 0.0014; mean = 7.79 nmol/mL (95% CI [6.30–9.28]) and AgNano + BC (*p* = 0.044; mean = 6.44 nmol/mL (95% CI [5.00–7.89]) groups compared to BC (mean = 4.60 nmol/mL (95% CI [2.75–6.45]). None of the groups differed significantly from the CTR group.

## 4. Discussion

Increasing human exposure to AgNPs, coupled with the rising incidence of fertility issues among men, raises important questions regarding the potential relationship between male exposure to AgNPs and reproductive disorders. The present study aimed to investigate the potential protective role of BC dietary intervention against the adverse effects induced by intragastric exposure to AgNPs on steroid modulation in the testis of adult male rats.

Despite the beneficial anti-bacterial properties of AgNPs, much research has indicated their toxic effects in higher organisms [40]. The mechanism behind these adverse effects is complex and includes, in particular, oxidative stress, inflammation, and associated tissue damage. The toxicity of AgNPs largely depends on factors such as dosage, particle size, duration of exposure, and route of administration [41]. Considering that ingestion is one of the main routes of human exposure to AgNPs due to food processing and migration from food packaging materials, in this work, we investigated the effect of an anti-oxidant food additive, namely blackcurrant pomace (BC), on AgNPs-induced adverse effects in the male reproductive system.

The growing interest in nanomaterials, including silver nanoparticles (AgNPs), has led to their extensive incorporation into everyday consumer goods, such as food and dietary supplements. Among the possible exposure pathways, the gastrointestinal tract represents the primary route. However, assessing the actual dietary intake of AgNPs in humans remains challenging due to the limited availability of up-to-date data [42]. As reported by Fröhlich and Fröhlich [43], daily levels of AgNPs exposure in humans range from 20 to 80 µg. Certain groups—particularly younger consumers—may ingest substantially higher amounts due to the appealing antimicrobial effects of AgNPs, for example, through colloidal-silver-based supplements [44]. It should also be emphasised that most dietary exposure originates from beverages containing silver as the food additive E174, of which up to 20% may be in nanoparticulate form [45], in fish and seafood [46,47,48], in mushrooms [49], as well as from household water purification devices [50] and from the migration of silver from AgNP-coated packaging and food contact materials [51]. Animal studies provide a more comprehensive framework for toxicology. Notably, Kim et al. [25,26] established a no-observed-adverse-effect level (NOAEL) of 30 mg/kg bw/day and a lowest-observed-adverse-effect level (LOAEL) of 125 mg/kg bw/day for orally administered AgNPs in rats, following sub-chronic exposure. In line with these findings, the present study employed a dose of 30 mg/kg bw/day, consistent with the NOAEL threshold, to ensure a sufficient biological response without triggering overt systemic toxicity. This dose is also representative of higher-end, yet plausible, human exposure scenarios from consumer products and supplements [52]. Therefore, the dosing strategy applied in the present study not only aligns with previous toxicological benchmarks but also facilitates direct comparability with other oral AgNPs exposure studies investigating redox and endocrine effects in vivo.

Body weight and liver biochemical indices are commonly used parameters to assess non-specific general toxicity. Consistent with the findings from previous in vivo studies [26,53,54,55], our experiment also did not show any negative effects on final body weights or liver function parameters following the treatment with AgNPs. This confirms the absence of significant toxic effects at the tested dose of the nanomaterial. At the same time, we did not observe any changes in feed intake and animal weight gains due to the administration of BC, nor from the co-treatment of BC and AgNPs. This result is consistent with findings reported by other authors [56]. The oral administration of AgNPs to the animals did not affect the plasma or liver activity of ALT and AST, suggesting that there was no systemic or liver toxicity [57]. Similarly, in our hands, the supplementation of feed with BC, as well as the co-treatment of BC and AgNPs, also had no effect on liver function. However, many studies indicate that the anthocyanins have a protective effect on the liver and improve liver function [58].

In line with the lack of effect of the general health parameters, no adverse signs of reproductive systemic toxicity were observed. Relative testis weight (GSI) is considered a non-specific parameter for evaluating the reproductive toxicity of tested substances. In our study, the relative weight of the testes remained unchanged between animals exposed to AgNPs and control rats. This finding is consistent with a previous report by Nauroze et al. [59] regarding a mouse study, which examined AgNPs exposure (50 mg/kg for 60 days). However, a higher GSI value was observed by Dong et al. [24] in animals fed a diet containing BC compared to control and AgNPs-treated animals, suggesting an improvement in testis function due to the intake of polyphenolic compounds.

The predominant bioactive compounds in BC, anthocyanins, are well-known for their antioxidative and anti-inflammatory potential in the male reproductive system [60,61]. Thus, the intention of the present study was to investigate the effects of BC in mitigating the oxidative stress induced by AgNPs. In our experiment, the BC-containing diet resulted in decreased protein carbonyl groups in the testes, counteracting the pro-oxidant effect of AgNPs in the testes. The protein carbonyl (PC) derivatives are a marker of oxidative stress, and their higher level corresponds with increased oxidative stress, and tissue cellular and tissue damage [62]. We found the highest level of carbonylated proteins in the testes of animals treated with AgNPs, compared to rats exposed to BC and those in the AgNPs + BC group, indicating increased oxidative stress induced by AgNPs and the protective effect of BC. This confirms our previous observation that BC mitigates testicular oxidative stress induced by exposure to biodiesel exhaust [33]. In line with this, dietary intervention with curcumin and vitamin E compounds has been shown to reduce the excess carbonylation in thyroxine-induced testicular oxidative stress in rats [63].

Reproductive physiology encompasses complex biological processes, including the regulation of androgen synthesis and function, which is controlled by the hypothalamic–pituitary–gonadal axis (HPG). The LH stimulates LCs to produce and secrete androgens [64]. Wang et al. [65] demonstrated that adult Balb/c mice receiving 30 nm AgNPs (30 or 125 mg/kg bw) by gavage showed no significant changes in LH and T levels, whereas Shehata et al. [66] reported decreased serum LH and T levels after oral administration of 100 nm AgNPs (50 mg/kg bw) to Sprague–Dawley rats for 90 days, as compared to the control animals. Observed in our study, no significant changes in plasma LH concentration likely result from a large dispersion in the results for LH in all experimental groups. Thus, in our study, BC diet supplementation did not cause any changes in plasma LH concentration, nor did BC supplementation combined with AgNPs exposure. In contrast, Li et al. [67] demonstrated that a diet supplemented with cyanidin-3-O-glucoside (0.5% m/m), the most widespread natural anthocyanin in food, mitigated the effects induced by cadmium exposure and restored plasma LH levels.

Androgen synthesis in LCs is also regulated by LH. The present study revealed no changes in intratesticular androgens (T and DHT) levels; however, their concentration in plasma was decreased in the experimental groups compared to the control animals. The divergence between the reduced plasma levels of T and DHT and the weak changes in testicular concentrations observed in our study suggests that mechanisms beyond local steroidogenesis are involved. AgNPs, when administered orally, primarily target the liver as the primary organ for accumulating silver (in both ionic and particulate forms) [68,69]. The distribution of AgNPs affects hepatic androgen metabolism and the synthesis of transport proteins. This suggests that increased oxidative stress and the promotion of inflammation in this organ may modulate androgen transport. However, the results presented by other authors indicate a lack of changes in SHBG level [35,70]. The systemic transport of androgens to target cells is also regulated by their binding to sex hormone-binding globulin (SHBG) and albumin, which determines the fraction of bioavailable hormones [71]. Alterations in the expression of these proteins, for example, under the influence of NPs, may reduce circulating androgen levels [72]. Because our study did not analyse the SHBG and albumin levels in plasma, we cannot definitively determine their involvement in regulating hormone transport. The lack of differences in the level of androgens in the testis under the influence of AgNPs, observed in our study, with concomitant changes in the expression level of genes involved in androgen synthesis, may be explained by the local mechanisms in the steroidogenic cells, such as increased ROS production and decreased mitochondrial membrane potential, which can also be recognised as the factors contributing to lower peripheral androgen levels [73]. It is suggested that the oral exposure to AgNPs presented in our experiment revealed lower toxicity than in in vitro studies or after the direct administration of AgNPs. The results from the in vitro study obtained by Hu et al. [74] demonstrated that increased T secretion in R2C Leydig cells following the incubation with anthocyanins (cyanidin-3-glucoside, delphinidin-3-glucoside, pelargonidin-3-glucoside, and cyanidin-3,5-diglucoside) contributed to increased testosterone secretion. The T metabolism in LCs results in the production of DHT, controlled by SRD5A type 1, or leads to E2 synthesis, influenced by Aro. The findings from our previous studies indicated that AgNPs influence the T metabolism, leading to an increased conversion of T to DHT formation [35]. On the other hand, AgNPs also modulate Aro expression in Leydig cells [75]. Some anthocyanins, such as cyanidin-3-glucoside, can inhibit the formation of DHT from T, demonstrating anti-androgenic effects [76]. It cannot be ruled out that the low levels of DHT observed in the gonads of the animal groups administered with BC were caused by the inhibitory effect of these compounds on the SRD5A1 enzyme. However, this hypothesis is contradicted by the higher Srd5a1 gene expression found in the gonads of animals exposed to both AgNPs and BC, as compared to those exposed only to AgNPs or to control animals. We did not observe any changes in Aro protein levels or E2 levels in the testes of rats exposed to AgNPs. This result was similar to what we found in animals that underwent dietary intervention with BC [33]. The lack of change may be attributed to the moderate pro-oxidant effect of AgNPs in the gonads, which likely had a weak inhibitory effect on the Aro protein expression in mitochondria. These findings are in line with the suggestions of other authors regarding the inhibition of Aro in steroidogenic cells exposed to AgNPs [77].

This study also focused on the regulation of gene expression involved in steroidogenesis in Leydig cells. In comparison to control animals, exposure to AgNPs resulted in the down-regulation of *Cyp11a1* and *Cyp17a1* genes. This is in contrast to the results of Garcia et al. [78], who showed increased expression of *Cyp11a1* and *Hsd3b1* genes in mice following the intravenous administration of 14 nm of AgNPs (at 1 mg/kg bw for 12 days), translating to increased T secretion. The letter aligns with our results, as in our experiment, other genes related to steroidogenesis, such as *Lhcgr*, *Hmgcr*, *StAR*, *Cyp11a1*, *Hsd17b3*, and *Hsd3b3*, were up-regulated after exposure to AgNPs and BC.

The observed up-regulation of *Hmgcr* gene expression in animals receiving AgNPs and BC suggests a potential role of polyphenols in enhancing steroidogenesis. These results support the findings reported by other authors, who demonstrated that anthocyanins can up-regulate the genes of the T synthesis pathway in LCs and reverse the inhibitory effect induced by chemical gonadotoxins [79,80,81]. Our study revealed a moderate up-regulation of the *StAR* gene in the testes of rats treated with AgNPs and fed with a diet containing BC compared to control animals, while no significant changes were observed in the gene expression in the testis of rats exposed to AgNPs or BC alone. This implies that the polyphenolic compounds from BC may interplay with AgNPs to increase *StAR* expression in the testis. The results obtained in an in vitro study [51] demonstrated that in R2C Leydig cells, 50µM of cyanidin-3,5-diglucoside reduced mitochondrial oxidative stress induced by 2,2′-azobis(2-amidinopropane)-dihydrochloride and up-regulated the expression of StAR protein.

Silver nanoparticles (AgNPs), regardless of the route of administration, affect the testis, leading to an increased production of reactive oxygen species (ROS) [12]. The ionic or particulate forms of silver lead to disruption of the mitochondrial membrane potential. This exposure leads to mitochondrial damage in both somatic and germline cells and activates apoptotic and inflammatory pathways [82]. Within the gonads, the CYP11A1 enzyme (also referred to as P450scc) is located in the inner mitochondrial membrane and plays a critical role in the conversion of cholesterol to pregnenolone [83]. Exposure to AgNPs can lead to mitochondrial damage, disrupt the redox balance, and reduce cholesterol availability within mitochondria, as indicated by reduced StAR gene and protein expression. These changes may consequently impair the transcription and/or activity of gonadal steroidogenic enzymes [40,84]. Conversely, anthocyanins possess the capability to scavenge ROS, enhance the activity of anti-oxidant defence enzymes, such as superoxide dismutase, catalase, and glutathione peroxidase [85], and stimulate the transcription factor Nrf2 [86]. These actions mitigate oxidative stress, improve the integrity of mitochondrial membranes, and restore optimal conditions for testosterone biosynthesis [87,88]. Furthermore, polyphenols may facilitate improved cholesterol transport into mitochondria, thereby enabling normal or even compensatory induction of steroidogenic enzyme expression [89]. Observed in the present study, co-treatment of the animals with AgNPs and BC enhances the gene expressions of steroidogenic enzymes (*Cyp11a1*, *Hsd17b3*, *Hsd3b3*, and *Cyp19a1*).

**Figure 6:**
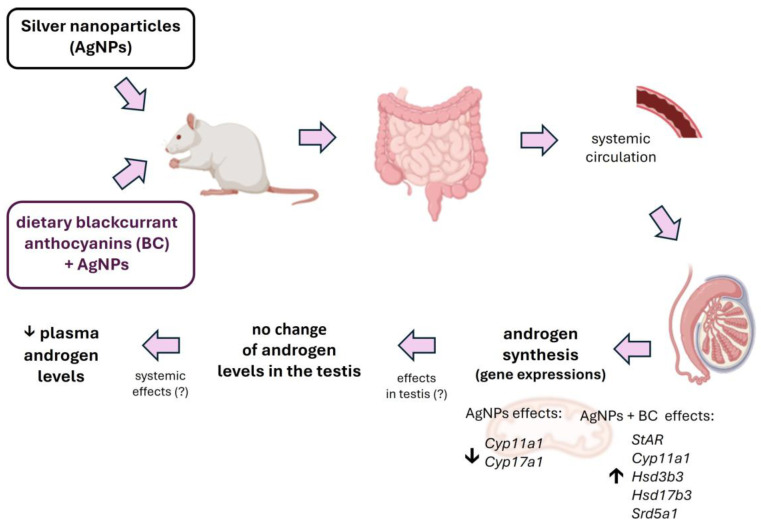
The effects of blackcurrant anthocyanins on AgNPs-induced oxidative stress in the testis. ↓,↑—decrease or increase.

Dietary anthocyanins from BC are proposed to protect the testes from AgNPs-induced damage directly by scavenging reactive oxygen species (ROS) and chelating Ag⁺ ions, and indirectly by activating endogenous anti-oxidant systems and enhancing antioxidative defence via increased levels of superoxide dismutase (SOD), catalase (CAT), glutathione peroxidase (GPx), and cellular glutathione (GSH), and finally by reducing lipid peroxidation, inflammation, and mitochondrial dysfunction, thereby restoring the expression and function of Leydig cell steroidogenic proteins (StAR, P450scc/CYP11A1, 3β/17β-HSD) essential for normal steroidogenesis [74,90,91,92]. Figure 6 presents the potential mechanism of the protective role of BC anthocyanins in AgNPs-induced oxidative stress in the testes.

Androgens/androgen receptor signalling are crucial for the normal development of the male reproductive system [93] and the maintenance of male sexual characteristics [94]. The current study showed higher expression of the Ar gene in the testes of animals repeatedly co-treated with AgNPs and BC compared to control animals. However, no significant effects on the Ar protein levels in the testes were observed in animals exposed to experimental factors. Our results suggest that dietary intervention with BC may strengthen the antioxidative protection mechanisms and indirectly affect the expression of the Ar gene in the testis. Such a suggestion was also proposed by Arisha et al. [95]. The authors reported that the increased oxidative stress induced in the testes of mice by repeated oral exposure to 20 nm AgNPs (50 mg/kg bw for 8 weeks) was partly reversed by morin (3,5,7,2′,4′-pentahydroxyflavone) co-treatment. In addition, the animals treated with AgNPs and morin were characterised by higher expression of the *Ar* gene in the testis, as compared to AgNP-treated animals.

Spermatogenesis is also regulated by oestrogens, as indicated by the presence of oestrogen receptors (ESR1 and ESR2) in various gonadal cells. ESR1 is primarily responsible for overseeing spermiogenesis, while ESR2 focuses on regulating spermatocyte apoptosis and the process of sperm release from the seminiferous epithelium in the testis. Activating oestrogen signalling can influence spermatogenesis, potentially resulting in reduced sperm output [96]. Observed in our study, a lack of changes in ESR1 protein level in the testis of experimental groups possibly suggests normal features of germ cell maturation. However, the reduced level of the ESR2 protein in the testis of rats from the AgNano + BC group, compared to the BC group, may indicate the susceptibility of testicular cells to oxidative damage. The exact role of anthocyanins in controlling E2/oestrogen receptor signalling is less well understood in relation to testis physiology. The results, based on in silico analysis performed by Nanashima et al. [22,88], confirmed the estrogenic activity of BC anthocyanins with a higher affinity for the ESR1 receptor than for the ESR2 receptor.

## 5. Conclusions

The findings from this study indicate that exposing animals to repeated doses of 30 mg/kg body weight of AgNPs via the gastrointestinal tract was associated with low general toxicity and limited effects on the reproductive system, as demonstrated by various parameters. Despite the relatively low toxicity of AgNPs to reproductive health, our research shows that this exposure is linked to increased oxidative stress in the gonads. Our investigations also revealed that the addition of 2% blackcurrant pomace into animals’ feed had an anti-oxidant effect and stimulated the expression of genes involved in steroid synthesis and metabolism, especially under pro-oxidant conditions induced by AgNPs. A 2% blackcurrant pomace supplementation in a rat diet is reasonably high but could be formulated into human functional foods. The bioactive compounds in blackcurrant pomace (especially polyphenols) have plausible mechanisms to influence reproductive health through anti-oxidant, anti-inflammatory, and gut-mediated pathways. However, translating findings from animal models to humans requires careful consideration of dose, bioavailability, and metabolism. Our results also revealed that the presence of AgNPs led to decreased signalling of the ESR2 gene in the testes, which may indirectly reflect a pro-oxidative state in the testis. These findings suggest that AgNP exposure may negatively impact testicular health and male fertility through oestrogen receptor-mediated pathways. The polyphenolic compounds may mitigate AgNP-induced oxidative stress, supporting their potential role in maintaining reproductive health.

Although we obtained interesting results, our study also had limitations. Our study examined the effects of a single dose of 20 nm nanosilver, without comparing other doses, varying nanoparticle sizes, or silver ions. This study also did not assess mitochondrial membrane potential or the Nrf2/HO-1 pathway, which somewhat limits the ability to link cellular morphological changes and biochemical markers to specific anti-oxidant defence mechanisms. Although rats are a commonly used model in in vivo studies of reprotoxicity, interspecies differences in nanoparticle distribution and sex hormone metabolism may account for differences in the nature and magnitude of the response. Therefore, caution is advised when attempting to extrapolate the results to other species, including humans.

## Figures and Tables

**Table 1:** Physical–chemical characterisation of AgNPs in water.

Parameter	BSA-Coated AgNPs
Nominal size of AgNPs [nm]	20 ± 5 ^#^
Size by dynamic light scattering [nm]	197.4 ± 2.7 ^#^
Polydispersity index	0.295 ± 4.2 ^#^
Zeta potential (mV)	−33.6 ± 3.5 ^#^
BET (Brunauer, Emmett, and Teller) surface area (m^2^/g)	2.2419
Micropore volume (cm^3^/g)	0.0076
Adsorption average pore width (nm)	13.6698
Desorption average pore width (nm)	23.8934

Data expressed as mean ± SD ^#^ (*n* = 3). Referenced from [29,30,31].

**Table 2:** The scheme of the in vivo experiment.

Experimental Factors:	Groups of Rats			
	CTR (*n* = 7)	AgNano (*n* = 7)	BC (*n* = 7)	AgNano + BC (*n* = 7)
rats treated with AgNPs	No	Yes	No	Yes
dietary intervention with BC (2% m/m added to standard animal feed)	No	No	Yes	Yes

**Table 3:** Primer sequences used for real-time PCR.

Gene	Forward Primer (5′-3′)	Reverse Primer (5′-3′)	Accession No.	Product Size (pb)
*Lhcgr*	TATATGCCCATCCCTGTTGG	ACCAAGACTGATCGCTCTGC	NM_013134.2	132
*Hmgcr*	AATGCCTTTGACAACCTCCTC	GGTTCGGATGCCTGTGTTAC	NM_012978.1	138
*StAR*	CGTCGGAGCTCTCTACTTGG	TTTCCTTCTTCCAGCCTTCC	NM_031558.3	139
*Cyp11a1*	TTGCCTTTGAGTCCATCACC	AGTCTGGAGGCATGTTGAGC	NM_017286.2	147
*Cyp17a1*	CCCAGATGGTGACTCAAAGC	CTCCAGTTTCTGGCCATCC	NM_012753.2	137
*Hsd17b3*	GGCTTTACCAGGGTCTTTCC	ACCTGTAGCTTTTCCAGTGTCC	NM_054007.1	150
*Hsd3b3*	TCAATCTGAAAGGTACCCAGAAC	TCATGATGCTCTTCCTCACG	NM_054007.1	145
*Cyp19a1*	CGTCATGTTGCTTCTCATCG	TACCGCAGGCTCTCGTTAAT	NM_017085.2	150
*Srd5a1*	GGATGGGAATCAACATCCAC	CAATAATCTCGCCCAGGAAA	NM_022711.4	132
*Ar*	GCGGAAGGGAAACAGAAGTA	CCCAGAGTCATCCCTGCTT	NM_012502.1	122
*Esr1*	AAAGAGAGTGCCAGGCTTTG	GCAAGTTAGGAGCAAACAGGA	NM_012689.1	143
*Esr2*	GTGCGTAGAAGGGATTCTGG	AGCCAAGGGGTACATACTGG	NM_012754.1	139
*Actb*	CTAAGGCCAACCGTGAAAAG	TCTCCGGAGTCCATCACAAT	NM_031144.3	136
*Gapdh*	GAGGACCAGGTTGTCTCCTG	ATGTAGGCCATGAGGTCCAC	NM_017008.4	161

**Table 4:** Food intake, final body weight, total weight gain, and gonadosomatic index (GSI) in rats.

Parameters	Rat Groups				
	CTR (*n* = 7)	AgNano (*n* = 7)	BC (*n* = 7)	AgNano + BC (*n* = 7)	ANOVA: *p*-Value; Effect Size (η_p_^2^)
Food intake [g/day]	17.1 ^A^ ± 0.3 ^B^ (16.3–17.9) ^C^	16.9 ± 0.2 (16.3–17.4)	16.3 ± 0.3 (15.6–17.0)	16.3 ± 0.3 (15.6–17.1)	*p* = 0.174; η_p_^2^ = 0.183
Final body weight [g]	328.5 ± 9.3 (305.8–351.2)	323.5 ± 5.3 (310.6–336.3)	314.0 ± 8.1 (294.2–333.8)	316.9 ± 4.1 (306.8–327.0)	*p* = 0.470; η_p_^2^ = 0.098
Total body weight gains [g]	24.7 ± 3.6 (15.9–33.4)	27.6 ± 8.8 (6.1–49.0)	20.2 ± 5.9 (5.7–34.7)	23.2 ± 2.3 (17.6–28.9)	*p* = 0.834; η_p_^2^ = 0.035
GSI [%]	0.95 ± 0.02 ^b^ (0.899–0.999)	0.95 ± 0.01 ^b^ (0.939–0.967)	1.01 ± 0.02 ^a^ (0.967–1.049)	0.98 ± 0.01 (0.941–1.012)	*p* = 0.043; η_p_^2^ = 0.283

Different superscripted lowercase letters (a, b) indicate statistically significant differences between experimental groups, at *p* < 0.05, as determined by one-way ANOVA with Duncan’s post hoc test; η_p_^2^—partial eta squared (effect size); ^A^—mean; ^B^—standard error of mean (S.E.M.); and ^C^—95% confidence interval (95% CI). CTR—control group; AgNano,—rats exposed to AgNPs (30 mg/kg/day for 28 days, by gavage; BC—rats receiving diet containing blackcurrant pomace (2% m/m); and AgNano + BC,—rats exposed to AgNPs and fed with diet containing blackcurrant pomace.

**Table 5:** ALT and AST activities in plasma and the liver in rats.

Parameters	Rat Groups				
	CTR (*n* = 7)	AgNano (*n* = 7)	BC (*n* = 7)	AgNano + BC (*n* = 7)	ANOVA: *p*-Value; Effect Size (η_p_^2^)
plasma ALT activity [U/mg protein]	0.46 ^A^ ± 0.06 ^B^ (0.301–0.624) ^C^	0.56 ± 0.06 (0.411–0.704)	0.64 ± 0.07 (0.448–0.833)	0.50 ± 0.04 (0.391–0.619)	*p* = 0.252 η_p_^2^ = 0.181
plasma AST activity [U/mg protein]	0.24 ± 0.05 (0.105–0.372)	0.20 ± 0.04 (0.109–0.299)	0.22 ± 0.04 (0.110–0.336)	0.28 ± 0.06 (0.122–0.445)	*p* = 0.699 η_p_^2^ = 0.067
ALT activity in the liver [U/mg protein]	3.77 ± 0.82 (1.67–5.87)	3.08 ± 0.22 (2.52–3.65)	2.36 ± 0.20 (1.86–2.86)	3.06 ± 0.82 (0.94–5.18)	*p* = 0.385 η_p_^2^ = 0.127
AST activity in the liver [U/mg protein]	7.71 ± 1.42 (4.24–11.20)	7.00 ± 0.72 (5.23–8.76)	5.39 ± 0.58 (3.97–6.81)	5.16 ± 0.90 (2.86–7.47)	*p* = 0.209 η_p_^2^ = 0.176

One-way ANOVA with Duncan’s post hoc test; η_p_^2^—partial eta squared; ^A^—mean; ^B^—standard error of mean (S.E.M.); and ^C^—95% confidence interval (95% CI). CTR—control group; AgNano—rats exposed to AgNPs (30 mg/kg/day for 28 days, by gavage; BC—rats receiving diet containing blackcurrant pomace (2% m/m); and AgNano + BC—rats exposed to AgNPs and fed with diet containing blackcurrant pomace (2% m/m).

## Data Availability

The raw data supporting the conclusions of this article will be made available by the authors on request.

## Supplementary Materials

The following supporting information can be downloaded at: https://www.mdpi.com/article/10.3390/nu17243809/s1. Table S1: The composition of selected phenolic compounds in experimental feeds. Table S2: Expression of mRNA of genes. References [97,98] are cited in the supplementary materials.

## Abbreviations

The following abbreviations are used in this manuscript:

AAPH	2,2′-azobis(2-amidinopropane)-dihydrochloride
Ag^+^	silver ion/ions
AgNPs	nanosilver/silver nanoparticles
ALT	alanine aminotransferase activity
ANOVA	one-way analysis of variance
AR/*Ar*	androgen receptor/androgen receptor gene
Aro	aromatase
AST	aspartate aminotransferase activity
BC	blackcurrant pomace
BSA	bovine serum albumin
*Cyp11a1*	cytochrome P450, family 11, subfamily a, gene
*Cyp17a1*	cytochrome P450, family 17, subfamily a, gene
*Cyp19a1*	P450 aromatase gene
DHT	dihydrotestosterone
E2	17β-estradiol
ELISA	enzyme-linked immunosorbent assay
ENPs	engineered nanoparticles
ESR1	oestrogen receptor type 1/α
ESR2	oestrogen receptor type 2/β
ESRs	oestrogen receptors
GIT	gastrointestinal tract
GSI	gonadosomatic index
*Hmgcr*	3-hydroxy-3-methylglutaryl-CoA reductase gene
HPG	hypothalamic-pituitary-gonadal axis
*Hsd3b3*	3 beta-hydroxysteroid dehydrogenase/delta5-4 isomerase type 3 gene
*Hsd17b3*	hydroxysteroid (17-beta) dehydrogenase 3 gene
i.g.	intragastrically
LCs	Leydig cells
LH	luteinizing hormone
*Lhcgr*/LHCGR	luteinizing hormone/choriogonadotropin receptor gene/protein
NPs	nanoparticles
NMs	nanomaterials
*Srd5a1*	steroid-5-alpha-reductase, alpha polypeptide 1 (3-oxo-5 alpha-steroid delta 4-dehydrogenase alpha 1) gene
PBS	phosphate-buffered saline
PVP-AgNPs	polyvinyl-pyrrolidone-coated silver nanoparticles
qPCR	quantitative polymerase chain reaction
*StAR*/StAR	steroidogenic acute regulatory protein gene/protein
T	testosterone
