# Supplementary material for: Anthocyanin-Rich Blackcurrant Pomace Mitigates Oxidative Stress and Affects Steroid Metabolism in the Testes of Rats Exposed to Silver Nanoparticles
Source: Nutrients. 2025 Dec 5;17(24):3809. doi: 10.3390/nu17243809 (PMC12735449; doi:10.3390/nu17243809)
Supplement: Supplementary file 1 [file nutrients-17-03809-s001.zip › nutrients-3968782-supplementary.pdf]

Supplementary materials

Anthocyanin-Rich Blackcurrant Pomace Mitigates Oxidative Stress and Affects Steroid Metabolism in the Testes of Rats Exposed to Silver Nanoparticles

Michał Oczkowski [1,*], Katarzyna Dziendzikowska [1], Marcin Kruszewski [2,3], Joanna Gromadzka-Ostrowska [1] and Agnieszka Grzelak [4]

[1] Department of Dietetics, Institute of Human Nutrition Sciences, Warsaw University of Life Sciences (SGGW-WULS), 159c Nowoursynowska St., 02-776 Warsaw, Poland; katarzyna_dziendzikowska@sggw.edu.pl (K.D.); joanna_gromadzka-ostrowska@sggw.edu.pl (J.G.-O.)

[2] Centre for Radiobiology and Biological Dosimetry, Institute of Nuclear Chemistry and Technology, 16 Dorodna Str., 03-195 Warsaw, Poland; m.kruszewski@ichtj.waw.pl

[3] Department of Molecular Biology and Translational Research, Institute of Rural Health, 2 Jaczewskiego Str., 20-090 Lublin, Poland

[4] Centre for Digital Biology and Biomedical Science-Biobank Lodz, Faculty of Biology and Environmental Protection, University of Lodz, 139 Pomorska Str., 91-402 Lodz, Poland; agnieszka.grzelak@biol.uni.lodz.pl

* Correspondence: michal_oczkowski@sggw.edu.pl; Tel.: +48-22-5937031

Table S1. The composition of selected phenolic compounds in experimental feeds page 2

Table S2. Expression of mRNA of genes page 4

Table S1. The composition of selected phenolic compounds in experimental feeds

Phenolic compound [mg/100g of animal feed]	Feed not supplemented with BC pomace	Feed supplemented with BC pomace
	[mg/100g of feed]	
gallic acid	n.d.*	0.56 ± 0.00
chlogenic acid	n.d.	0.71 ± 0.02
myricetin	n.d.	0.58 ± 0.01
quercetin	n.d.	0.27 ± 0.00
kaempferol	n.d.	0.04 ± 0.01
quercetin-3-*O*-rutinoside	n.d.	2.41 ± 0.01
quercetin-3-*O*-glucoside	n.d.	1.08 ±0.01
cyanidin-3.5-di-*O*-rutinoside	n.d.	20.28 ± 0.11
delphinidin-3.5-di-*O*-rutinoside	n.d.	27.51 ± 0.12
delphinidin-3.5-di-*O*-glucoside	n.d.	8.26 ± 0.03
cyanidin-3.5-di-*O*-glucoside	n.d.	18.42 ± 0.02

*n.d. – not detected; Referenced from

The determination of selected phenolics (gallic and chlorogenic acids), flavonols (myricetin, quercetin, kaempferol, quercetin-3-O-rutinoside, and quercetin-3-O-glucoside), and anthocyanidins (cyanidin-3,5-di-O-rutinoside, delphinidin-3,5-di-O-rutinoside, delphinidin-3,5-di-O-glucoside, and cyanidin-3,5-di-O-glucoside) in experimental feeds was made following the procedure outlined by Hallmann et al. [1] and Dóka et al. [2] For the analysis of phenolic acids and flavonoids, the mobile phases were composed of acetonitrile (10%, v/v) and ultrapure deionised water (55%, v/v). Phenolic acids and flavonoids were detected at wavelengths of 280 nm and 340 nm, respectively. Polyphenol content was determined in triplicate, and five injections of external standards (99.0% purity) were used. Anthocyanins were eluted from a 1 mL extract using an isocratic flow and a mobile phase composed of 5% acetic acid, acetonitrile, and methanol (70:10:20, v/v/v) at a flow rate of 1.5 mL/min. Detection occurred at 530 nm. Individual anthocyanins were identified using pure standards (99.9% purity) and retention times obtained from chromatograms.

References:

1. Hallmann, E.; Kazimierczak, R.; Marszałek, K.; Drela, N.; Kiernozek, E.; Toomik, P.; Matt, D.; Luik, A.; Rembiałkowska, E. The Nutritive Value of Organic and Conventional White Cabbage (Brassica Oleracea L. Var. Capitata) and Anti-Apoptotic Activity in Gastric Adenocarcinoma Cells of Sauerkraut Juice Produced Therof. *J. Agric. Food Chem.* **2017**, 65, 37, 8171-8183. https://doi.org/10.1021/acs.jafc.7b01078.

2. Dóka, O.; Ficzek, G.; Bicanic, D.; Spruijt, R.; Luterotti, S.; Tóth, M.; Buijnsters, J.G.; Végvári, G. Direct Photothermal Techniques for Rapid Quantification of Total Anthocyanin Content in Sour Cherry Cultivars. *Talanta* **2011**, *84, 2, 341-346*. https://doi.org/10.1007/s10967-012-2320-y.

Table S2. Expression of mRNA of genes.

gene	BC *vs* CTR			AgNPs *vs* CTR			AgNPs+BC *vs* CTR		
	expression	95% C.I.	Result	expression	95% C.I.	Result	expression	95% C.I.	Result
Lhcgr	0.832	0.445 - 1.575	-	0.607	0.233 - 1.618	-	**1.969**	**1.333 - 3.246**	**up**
Hmgcr	1.302	0.736 - 2.051	-	1.235	0.594 - 2.915	-	**2.647**	**1.558 - 4.176**	**up**
StAR	0.889	0.593 - 1.482	-	0.978	0.679 - 1.777	-	**1.904**	**1.455 - 3.010**	**up**
Cyp11a1	1.066	0.784 - 1.656	-	**0.408**	**0.212 - 0.675**	**down**	**1.683**	**1.167 - 2.593**	**up**
Cyp17a1	1.080	0.365 - 2.722	-	**0.388**	**0.170 - 0.813**	**down**	1.650	0.577 - 4.949	-
Hsd17b3	1.169	0.725 - 1.801	-	0.837	0.482 - 1.317	-	**2.510**	**1.667 - 3.355**	**up**
Hsd3b3	1.272	0.914 - 1.609	-	0.955	0.703 - 1.218	-	**2.312**	**1.619 - 3.095**	**up**
Cyp19a1	1.387	0.717 - 2.955	-	1.215	0.887 - 2.066	-	**1.669**	**1.166 - 2.751**	**up**
Srd5a1	1.048	0.735 - 1.649	-	1.099	0.790 - 1.781	-	**2.441**	**2.068 - 3.227**	**up**
Ar	**1.356**	**1.012 - 2.103**	**up**	1.219	0.996 - 1.576	-	**2.684**	**1.996 - 3.671**	**up**
Esr1	1.118	0.766 - 1.852	-	1.126	0.912 - 1.412	-	**2.453**	**1.954 - 3.178**	**up**
Esr2	**1.866**	**1.102 - 3.116**	**up**	0.833	0.496 - 1.220	-	**1.873**	**1.169 - 2.680**	**up**

Expression is presented as a fold change of expression relative to the control (Ctr) group. Significance was tested *via* Pairwise Fixed Reallocation Randomisation Test using REST 2009 software. *ACTB* and *GAPDH* were used as reference genes.
